# Intracardiac Neoplastic Extension of a Malignant Solitary Fibrous Tumor Mimicking a Giant Left Atrial Thrombus

**DOI:** 10.7759/cureus.113219

**Published:** 2026-07-23

**Authors:** Samia Benlabed, Giovanni Cuminetti, Frederic Vanden Eynden, Constantin Stefanidis

**Affiliations:** 1 Cardiology, Erasmus Hospital, Bruxelles, BEL; 2 Cardiology, Chirec Delta, Bruxelles, BEL; 3 Cardiac Surgery, Erasmus Hospital, Bruxelles, BEL

**Keywords:** echocardiography, left atrial thrombus, malignant solitary fibrous tumor, mobile cardiac thrombus, surgical thrombectomy

## Abstract

Intracardiac masses in patients with advanced malignancy pose a major diagnostic and therapeutic challenge, as neoplastic intravascular extension can closely mimic thrombus. We report a patient with metastatic malignant solitary fibrous tumor (SFT) who presented with a giant multilobulated left atrial mass (>5 cm) initially suspected to represent thrombus. Transthoracic and transesophageal echocardiography demonstrated a highly mobile mass prolapsing through the mitral valve into the left ventricle during diastole, with no right-sided involvement, no significant valvular pathology, and no history of atrial fibrillation or structural heart disease. Given the imminent risk of systemic embolization or mitral valve obstruction, therapeutic anticoagulation was started, and the mass was surgically resected after multidisciplinary discussion.

Histopathology demonstrated an intravascular malignant neoplasm of epithelioid cells, with severe nuclear atypia, brisk mitotic activity, and a hemangiopericytoma-like vascular pattern; tumor cells were STAT6-positive, confirming metastatic malignant SFT. This case illustrates the diagnostic difficulty of distinguishing thrombus from neoplastic intravascular extension in cancer patients and underscores the complementary roles of echocardiography and histopathological confirmation in guiding urgent, multidisciplinary management of highly mobile left atrial masses.

## Introduction

Left atrial masses are uncommon and most frequently represent thrombi in patients with atrial fibrillation, mitral valve disease, or severe left atrial enlargement [1]. Giant mobile masses prolapsing through the mitral valve are particularly rare and present a high risk of systemic embolization and acute hemodynamic compromise [2]. 

Patients with cancer have an increased risk of intracardiac masses due not only to cancer-associated hypercoagulability but also to direct neoplastic intravascular extension [3,4]. Distinguishing between thrombotic and neoplastic lesions is clinically crucial, as therapeutic strategies and prognostic implications differ significantly. Imaging findings may overlap considerably, particularly in highly mobile intracardiac lesions [5]. 

Solitary fibrous tumors (SFTs) are rare mesenchymal neoplasms characterized by the NAB2-STAT6 fusion gene and nuclear STAT6 expression on immunohistochemistry. Although initially described as pleural tumors, SFTs may arise in nearly any anatomical location and exhibit variable biological behavior, ranging from indolent lesions to highly aggressive metastatic malignancies [6,7]. Cardiac involvement by SFT is exceptionally rare. 

Approximately 10%-20% of SFTs behave malignantly and may spread by local invasion, hematogenous dissemination, or direct intravascular extension through adjacent venous structures such as the pulmonary veins [7-9]. This mechanistic diversity, combined with the nonspecific echocardiographic appearance of intracavitary tumor extension, makes distinguishing neoplastic involvement from bland thrombus particularly challenging on imaging alone, as illustrated by the present case. 

In patients with advanced malignancy, surgical management of intracardiac masses may be complicated by overall prognosis, making conservative approaches often preferable. However, giant highly mobile masses prolapsing through the mitral valve represent a surgical emergency because of the imminent risk of embolization or mechanical obstruction [2]. 

We report a case of a giant multilobulated left atrial neoplastic mass in a patient with metastatic malignant SFT, detailing the echocardiographic features, pathological findings, clinical decision-making, and therapeutic management. 

## Case presentation

The patient was a 63-year-old woman with a known primary malignant SFT arising in the left anterior mediastinum, complicated by multiple pulmonary and muscular metastases. SFTs are rare mesenchymal neoplasms characterized by NAB2-STAT6 gene fusion, with a subset demonstrating aggressive metastatic behavior [6-8]. There was no history of atrial fibrillation, significant valvular heart disease, or known structural cardiac abnormalities. 

**Figure 1:**
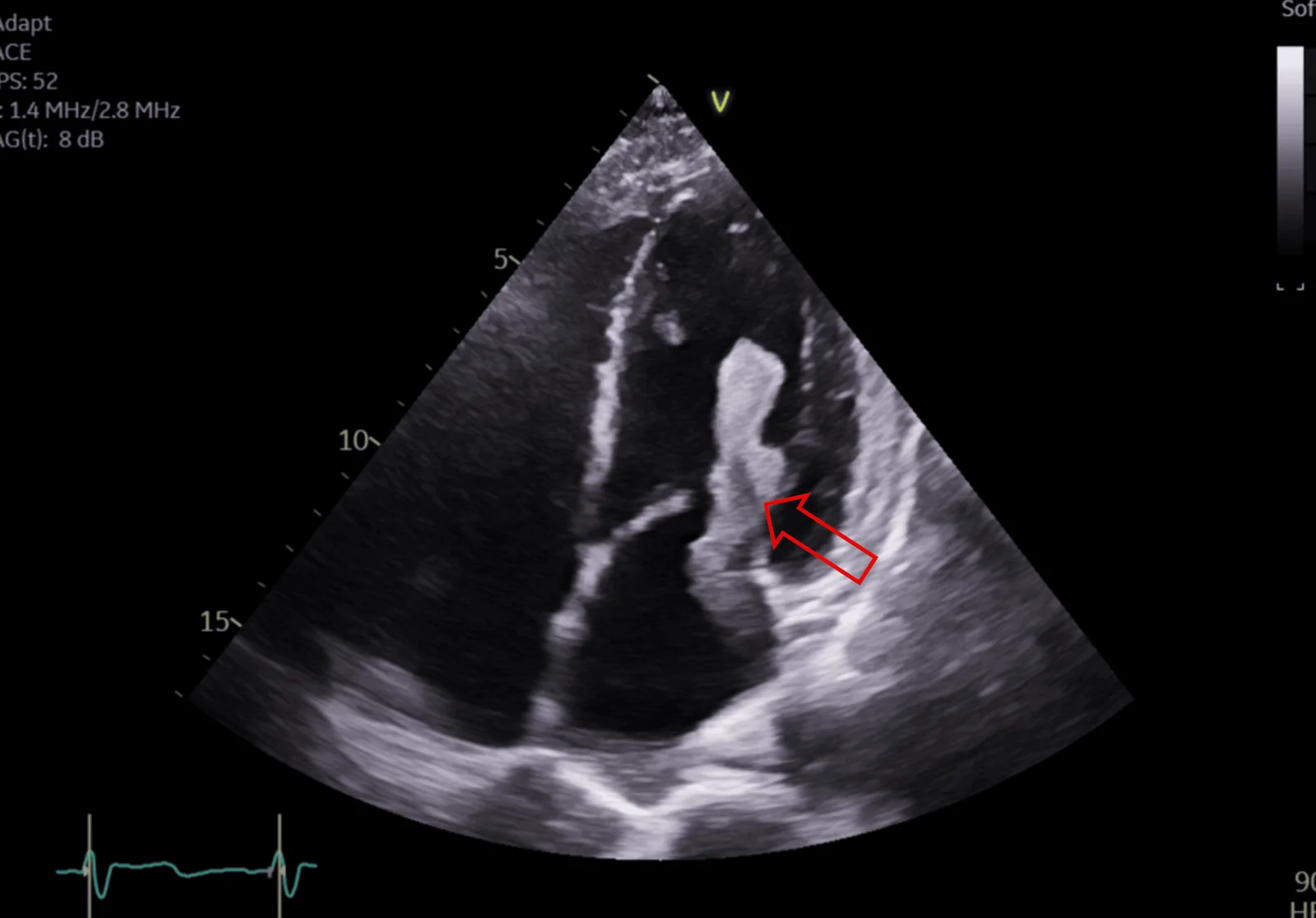
Transthoracic echocardiography (apical four-chamber view), demonstrating a giant multilobulated left atrial mass prolapsing into the left ventricle.

Transthoracic echocardiography revealed a giant multilobulated mobile left atrial mass (>5 cm), floating freely and prolapsing through the mitral valve during diastole, with protrusion toward the left ventricular apex, followed by complete retraction into the atrium during systole (Figure 1). No mass was observed in the right-sided cardiac chambers, and the cardiac valves were structurally normal. 

**Figure 2:**
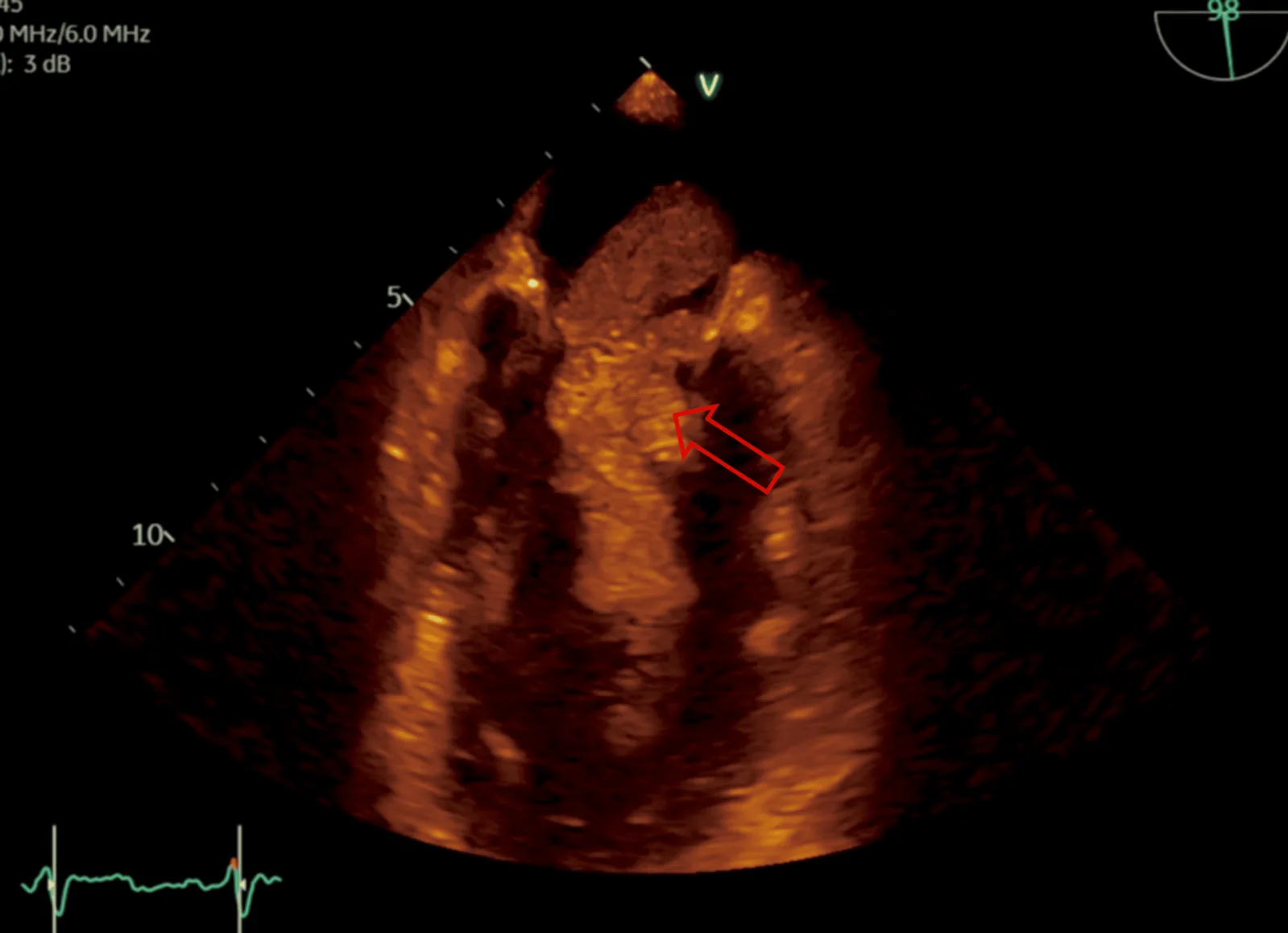
Transesophageal echocardiography (mid-esophageal two-chamber view), demonstrating a giant multilobulated left atrial mass prolapsing into the left ventricle.

Transesophageal echocardiography was performed for further anatomical characterization and confirmed a highly mobile multilobulated left atrial mass, with dynamic transmitral prolapse, absence of significant valvular pathology, and absence of right-sided intracardiac involvement (Figure 2). No dedicated computed tomography or cardiac magnetic resonance imaging was obtained prior to surgery, given the urgency of the presentation; diagnostic assessment relied on transthoracic and transesophageal echocardiography alone, a limitation discussed further below. 

The extreme mobility of the lesion and its repetitive prolapse through the mitral valve throughout the cardiac cycle placed the patient at an exceptionally high risk for systemic embolization or acute mitral valve obstruction. 

Therapeutic anticoagulation was immediately initiated, given the presumed thromboembolic risk pending definitive management. Although surgical intervention carries elevated risk in patients with metastatic malignancy, the imminent threat of catastrophic embolization or acute mitral valve obstruction necessitated urgent surgical management. 

**Figure 3:**
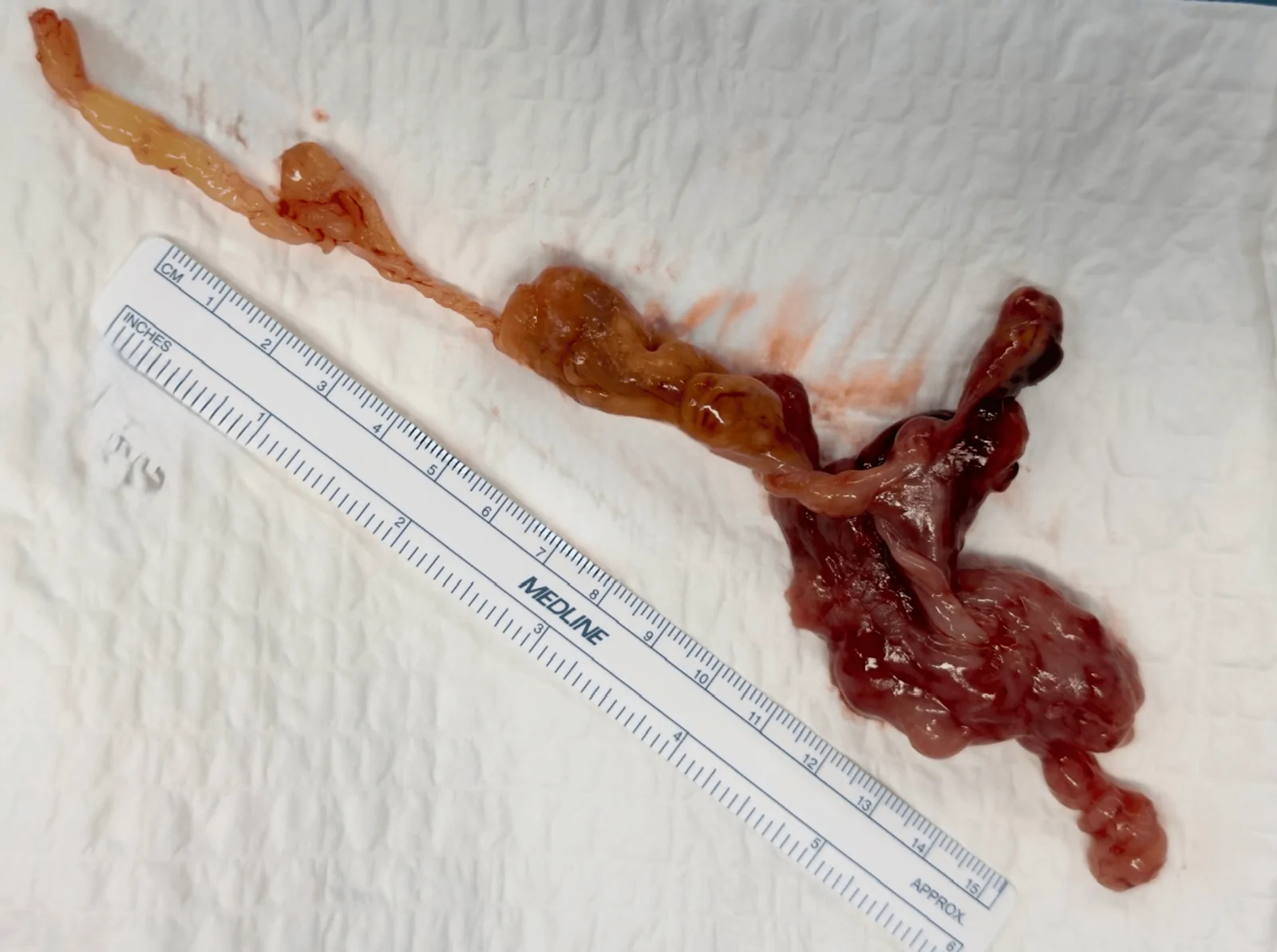
Surgical specimen of the extracted intracardiac mass.

A multidisciplinary discussion involving cardiology, cardiac surgery, oncology, imaging specialists, and pathology confirmed that surgical resection was indicated despite the patient's advanced oncologic disease burden. Given the tumor's transvenous extension, resection required a combined surgical approach through the aortopulmonary window, together with left atriotomy. Surgical thrombectomy/resection was successfully performed (Figure 3). 

Surgical pathology comprised two specimens. The first, from the aortopulmonary window, consisted of a fibro-adipose fragment measuring 1 x 0.5 x 0.2 cm, submitted in toto; an associated lymph node was without particularity. The second specimen, representing invasion of the left atrium through the left superior pulmonary vein, was a fleshy fragment measuring 7.5 x 4.5 x 2 cm, sampled in serial sections. 

**Figure 4:**
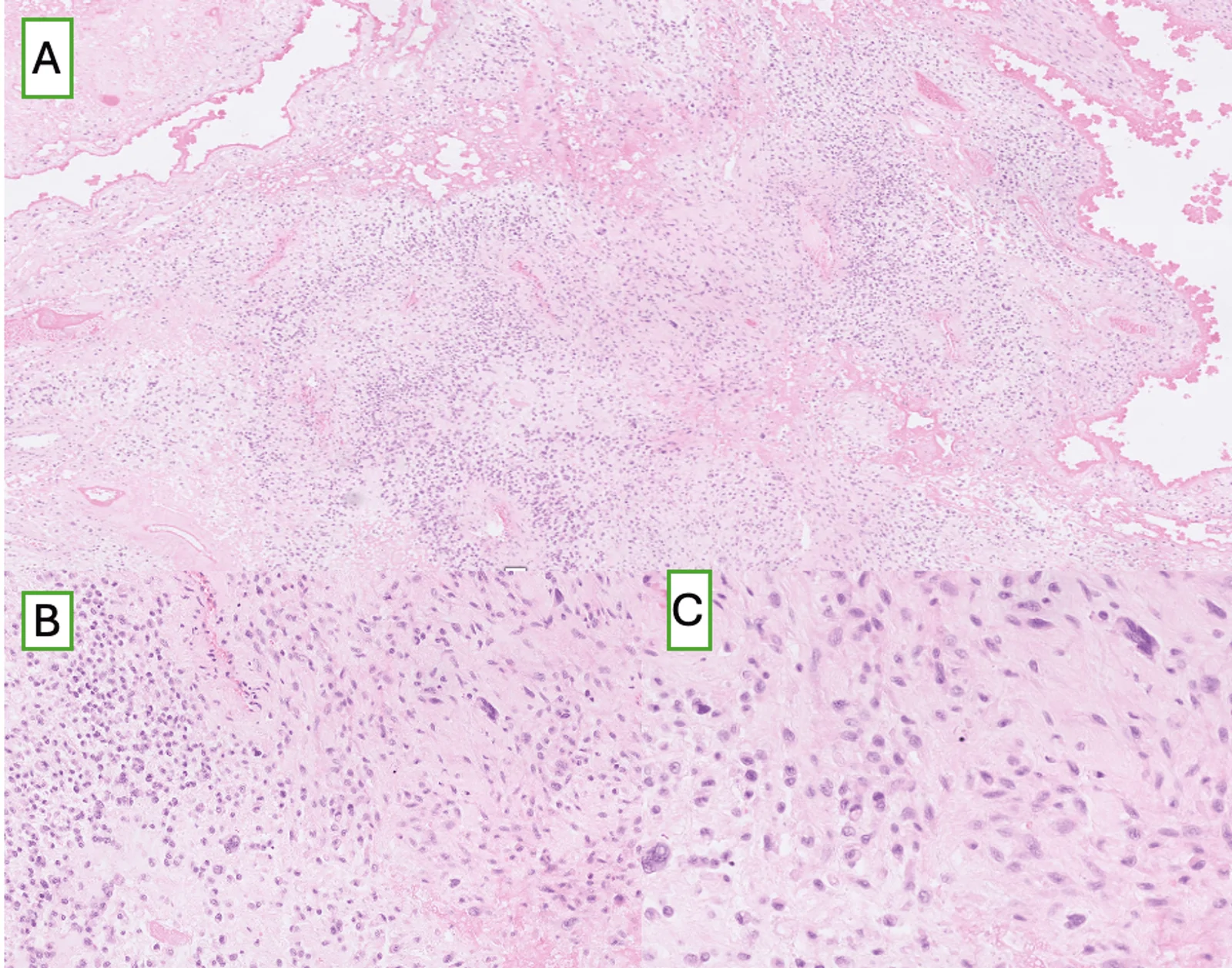
Hematoxylin and eosin staining, demonstrating intravascular localization of an undifferentiated malignant neoplasm composed of epithelioid cells (A: x2.5; B: x10; C: x20).

Histopathological examination demonstrated intravascular localization of an undifferentiated malignant neoplasm composed of epithelioid cells with nonspecific morphology, severe nuclear atypia, and numerous mitotic figures; a formal quantitative mitotic count was not performed. Tumor vascularization was abundant and exhibited a characteristic hemangiopericytoma-like appearance (Figure 4). 

**Figure 5:**
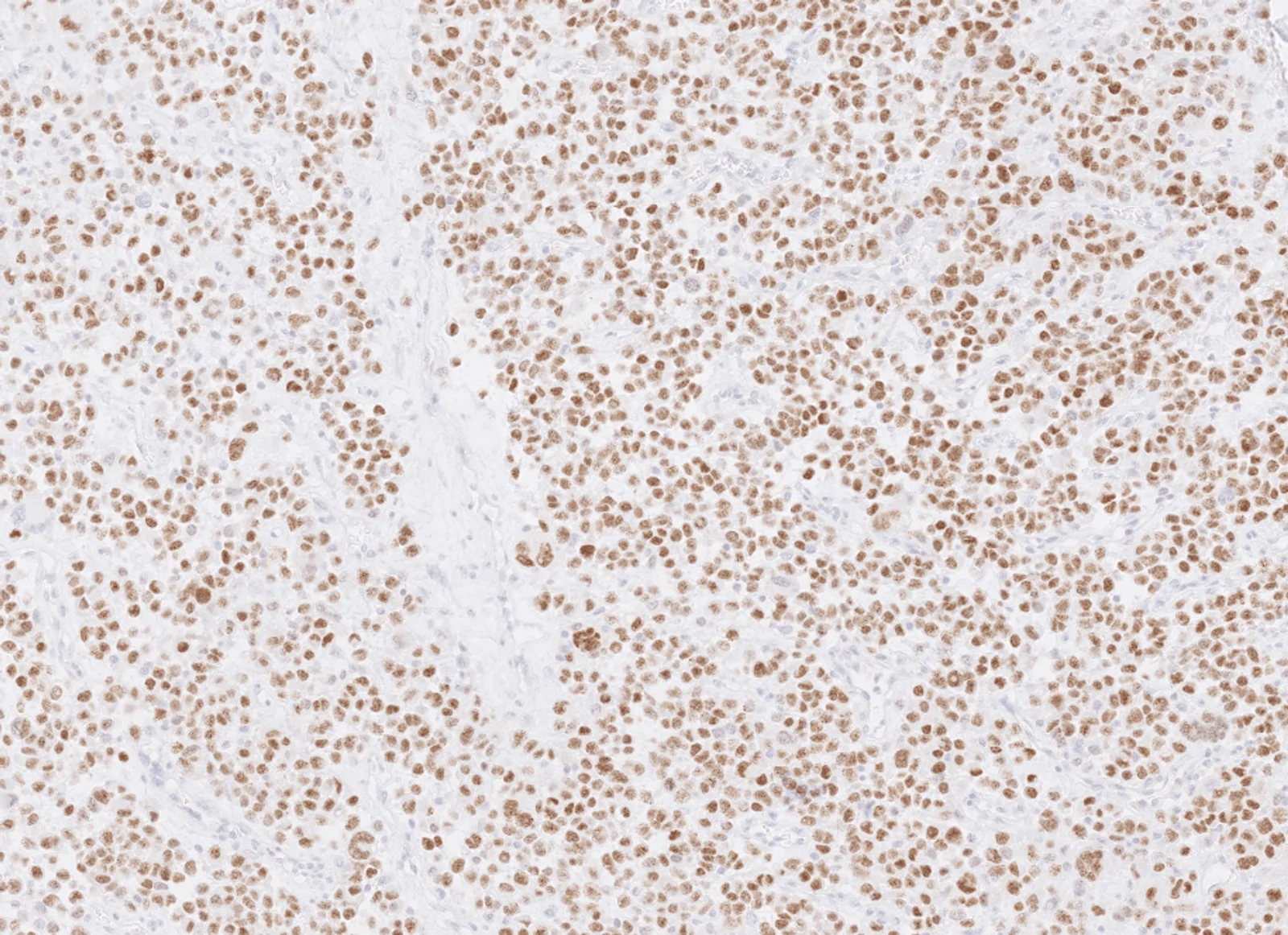
STAT6 immunohistochemistry, demonstrating diffuse nuclear positivity in tumor cells.

Immunohistochemical analysis demonstrated diffuse strong nuclear STAT6 positivity in tumor cells (Figure 5). Additional immunohistochemical markers frequently reported in SFT (CD34, BCL-2, CD99) and the Ki-67 proliferation index were not assessed, and margin status was not formally reported; this represents a limitation of the pathological workup, discussed further below. 

The pathological analysis found an undifferentiated malignant tumor with intravascular localization, extending from the left superior pulmonary vein into the left atrium, whose immunohistochemical profile (STAT6+) is consistent with the patient's known history of malignant SFT. 

These findings established the diagnosis of intracardiac neoplastic involvement, via direct intravascular extension through the left superior pulmonary vein, by metastatic malignant SFT rather than bland thrombus formation. 

## Discussion

Giant mobile left atrial masses are rare and carry exceptionally high embolic and obstructive risk. Classically, highly mobile left atrial masses are associated with thrombi occurring in the context of atrial fibrillation, rheumatic mitral stenosis, or severe left atrial enlargement [1,2]. 

Unlike classic left atrial thrombi, the present case demonstrated the absence of atrial fibrillation, the absence of significant mitral valve disease, the absence of marked left atrial enlargement, and a known metastatic malignant disease. Although the echocardiographic appearance initially suggested giant thrombus formation, histopathological analysis ultimately demonstrated neoplastic intravascular extension of metastatic malignant SFT. This distinction is clinically essential because imaging findings alone may not reliably differentiate thrombotic from neoplastic intracardiac lesions. Both entities may exhibit high mobility, multilobulated morphology, intracavitary extension, and dynamic prolapse through cardiac valves. 

Definitive diagnosis therefore frequently requires histopathological examination. 

SFTs are uncommon mesenchymal neoplasms with variable biological behavior [6-8]. While many SFTs follow an indolent clinical course, malignant forms may demonstrate local recurrence, distant metastases, vascular invasion, and aggressive progression [7-9]. 

Cardiac involvement by SFT is exceptionally rare, with only a small number of cases reported in the literature [5,10]. Potential mechanisms include hematogenous dissemination, direct intravascular extension, and metastatic intracardiac implantation [5]. In the present case, extension occurred by direct intravascular growth through the left superior pulmonary vein into the left atrium, a mechanism also described in previously reported cases (see comparison below). 

The presence of STAT6 positivity strongly supports the diagnosis of SFT and represents a highly sensitive and specific immunohistochemical marker for these tumors [6]. The marked hemangiopericytoma-like vascular pattern observed in this case is also characteristic of SFT and supports the pathological diagnosis [8]. 

Intracardiac extension of malignant SFT has been described in only a handful of prior reports, most involving direct transvenous extension into the left atrium via a pulmonary vein, though hematogenous spread to multiple chambers has also been reported. Table 1 summarizes previously published cases alongside the present report. 

**Table 1:** Comparison of previously reported cases of malignant solitary fibrous tumor with intracardiac extension LA: left atrium, LV: left ventricle, RV: right ventricle. The present case is highlighted.

Study	Age/sex	Primary site	Route of cardiac extension	Chamber(s) involved	Treatment	Outcome
Cuadrado et al. [11]	74, F	Pleura	Intracavitary extension via the left upper pulmonary vein	LA	Surgical resection of the primary tumor	Not detailed in report
Hashmi et al. [12]	67, F	Pleura (recurrent)	Invasion of the left inferior pulmonary vein into the LA	LA	Pneumonectomy + LA mass resection, with pericardial patch	Died in-hospital, day 107 (multiorgan complications)
Suzuki et al. [13]	24, F	Forearm (soft tissue), then pleura	Left atrial endocardium (mechanism not specified)	LA	Resection of forearm, pleural, and LA tumors	Not detailed in report
Alonso et al. [14]	87, M	Pleura	Hematogenous (multiple chambers)	RV free wall, interventricular septum, LV free wall	None (systemic metastatic disease)	Died (massive pulmonary thromboembolism)
This study	63, F	Left anterior mediastinum	Intravascular extension via the left superior pulmonary vein	LA	Surgical resection (aortopulmonary window approach + LA tumor resection)	Not available

As illustrated in Table 1, direct transvenous extension into the left atrium via a pulmonary vein, as in the present case, is the most frequently reported route, though the interval between diagnosis of the primary tumor and cardiac involvement, as well as outcomes, varied considerably across reports, ranging from surgical resection with short-term survival to rapid postoperative or disease-related death. This variability underscores the aggressive and unpredictable natural history of malignant SFT once cardiac extension occurs. 

The differential diagnosis of a giant, mobile left atrial mass includes left atrial thrombus, cardiac myxoma, primary cardiac sarcoma, tumor thrombus (e.g., renal cell carcinoma extending via the inferior vena cava), and metastatic extension through the pulmonary veins, as occurred in this case [5,15]. Left atrial thrombus is by far the most common etiology and is favored by a background of atrial fibrillation, mitral stenosis, or marked atrial enlargement, none of which were present here. Cardiac myxoma typically arises as a pedunculated mass with a discrete stalk attached to the region of the fossa ovalis and tends to be more homogeneous in echotexture. Primary cardiac sarcomas are usually broad-based, heterogeneous, and may show hypoechoic areas corresponding to necrosis, with a propensity for local invasion rather than a stalk. Tumor thrombus and metastatic transvenous extension, as in the present case, are suggested by direct continuity between the mass and an adjacent venous structure, though this continuity can be difficult to appreciate on two-dimensional echocardiography alone [15]. 

In this case, preoperative diagnosis remained difficult because the absence of atrial fibrillation and mitral valve disease argued against a classic thrombus without excluding a neoplastic one, and because extreme mobility with multilobulated morphology can be seen with both thrombus and tumor. No dedicated cross-sectional imaging (computed tomography/magnetic resonance imaging) was obtained, given the urgency of the presentation, and the known history of metastatic SFT raised suspicion for neoplastic involvement without confirming a cardiac origin in the absence of tissue diagnosis. Ultimately, only histopathological examination, which revealed the transvenous route of extension and STAT6 positivity, established the definitive diagnosis. 

Echocardiography was central to the detection of the intracardiac lesion, the characterization of size and mobility, the assessment of mitral valve interaction, the evaluation of embolic and obstructive risk, and the guidance of therapeutic decision-making. Transesophageal echocardiography provided superior anatomical resolution and allowed detailed evaluation of the lesion morphology, the dynamic transmitral prolapse, the intracardiac extension, and the absence of associated valvular disease. 

The repetitive prolapse of the mass through the mitral valve created a physiology similar to intermittent “ball-valve” obstruction, potentially capable of causing sudden hemodynamic collapse. 

Management of intracardiac neoplastic masses remains challenging, particularly in patients with advanced metastatic malignancy. Potential therapeutic options include anticoagulation, surgical resection, systemic oncologic therapy, or palliative management. 

A giant, highly mobile, multilobulated echodense mass prolapsing through the mitral valve is the classic echocardiographic description of a ball-valve left atrial thrombus, and given that thrombus is statistically far more common than neoplastic intracavitary extension, it was reasonably considered the leading initial diagnosis. Empiric therapeutic anticoagulation was instituted immediately, as is standard first-line management for any newly identified highly mobile intracavitary mass with embolic potential, since delaying treatment while awaiting definitive characterization would itself have carried embolic risk. 

Because the tumor extended transvenously through the left superior pulmonary vein, operative resection required a combined surgical exposure through the aortopulmonary window together with left atriotomy to achieve removal of both the intracavitary and venous components. Resection in the setting of known widespread metastatic disease also required balancing complete oncologic clearance against operative risk in a patient with limited overall prognosis. 

Because thrombus and neoplastic intravascular extension can be clinically and echocardiographically indistinguishable, and because their management differs fundamentally, histopathological confirmation was considered essential before any long-term therapeutic strategy could be defined; surgery therefore served the dual purpose of definitive treatment and diagnostic confirmation. 

In the present case, urgent surgical intervention was favored because the lesion was highly mobile with an imminent embolic risk, a possible acute mitral inflow obstruction, and a sudden cardiovascular death risk. Therapeutic decision-making required balancing the operative risk, the metastatic disease burden, the oncologic prognosis, and the immediate cardiovascular danger. 

This case emphasizes the importance of multidisciplinary collaboration involving cardiology, cardiac surgery, oncology, imaging specialists, and pathology. 

This report has several limitations. Postoperative imaging surveillance, recurrence status, adjuvant oncologic therapy, and long-term survival data were not available at the time of manuscript preparation, precluding assessment of recurrence risk and long-term outcome, both of particular importance given the recognized potential for local recurrence and metastasis in malignant SFT [7,8]. 

Preoperative diagnostic workup was limited to transthoracic and transesophageal echocardiography; cross-sectional imaging (computed tomography/magnetic resonance imaging) and dedicated spectral Doppler assessment of the mass were not performed, given the urgency of the clinical presentation, and could have provided additional information regarding tissue characterization, vascular continuity with the pulmonary vein, and enhancement pattern [15]. 

Finally, the immunohistochemical panel was limited to STAT6; additional markers supportive of SFT and of prognostic value in malignant variants (CD34, BCL-2, CD99, Ki-67 proliferation index) were not assessed, and a formal quantitative mitotic count and margin status were not reported. Future studies and case reports would benefit from more complete pathological characterization and structured long-term follow-up to better define the natural history of intracardiac extension of malignant SFT. 

## Conclusions

Intracardiac extension of malignant SFT is exceptionally rare and may mimic giant left atrial thrombus on echocardiography. Histopathological examination remains essential for definitive diagnosis, particularly in patients with known metastatic malignancy. Highly mobile left atrial neoplastic masses prolapsing through the mitral valve represent a medical and surgical emergency because of the risk of catastrophic systemic embolization and acute mitral inflow obstruction. 

This case underscores the diagnostic limitations of imaging alone, the importance of pathological confirmation, the critical role of echocardiography in risk stratification, and the necessity of multidisciplinary management in complex cardio-oncologic presentations. Multimodality imaging, where feasible, combined with histopathological confirmation and multidisciplinary management, remains central to diagnosis and treatment, and long-term surveillance is warranted, given the recognized recurrence potential of malignant SFTs. 
